# Exploring the potential of propanamide-sulfonamide based drug conjugates as dual inhibitors of urease and cyclooxygenase-2: biological and their *in silico* studies

**DOI:** 10.3389/fchem.2023.1206380

**Published:** 2023-08-03

**Authors:** Saghir Ahmad, Muhammad Abdul Qadir, Mahmood Ahmed, Muhammad Imran, Numan Yousaf, Tanveer A. Wani, Seema Zargar, Ijaz Ali, Muhammad Muddassar

**Affiliations:** ^1^ School of Chemistry, University of the Punjab, Lahore, Pakistan; ^2^ Department of Microbiology, Immunology and Cancer Biology, School of Medicine, University of Virginia, Charlottesville, VA, United States; ^3^ Department of Chemistry, Division of Science and Technology, University of Education, Lahore, Pakistan; ^4^ KAM School of Life Sciences, FC College (A Chartered University), Lahore, Pakistan; ^5^ Department of Biosciences, COMSATS University Islamabad, Islamabad, Pakistan; ^6^ Department of Pharmaceutical Chemistry, College of Pharmacy, King Saud University, Riyadh, Saudi Arabia; ^7^ Department of Biochemistry, College of Science, King Saud University, Riyadh, Saudi Arabia; ^8^ Center for Applied Mathematics and Bioinformatics, Gulf University for Science and Technology, Kuwait City, Kuwait

**Keywords:** sulfonamides, NSAIDs, urease, inflammation, cyclooxygenase-2, *in-silico* studies

## Abstract

Derivative synthesis has been a crucial method for altering the effects of already-approved medications, especially to lessen adverse effects and enhance results. Making use of this multi-target approach, a series of naproxen-sulfa drug conjugates was designed and synthesized. The newly designed conjugates were confirmed by spectroscopic techniques like IR, ^1^HNMR, ^13^CNMR, and elemental analysis. The conjugates were screened for anti-inflammatory, urease, and cyclooxygenase-2 (COX-2) inhibition. Naproxen conjugated with sulfanilamide, sulfathiazole, and sulfaguanidine was found potent and showed a competitive mode of urease inhibition, with IC_50_ (µM) values 6.69 ± 0.11, 5.82 ± 0.28, 5.06 ± 0.29, respectively. When compared to other screened conjugates, the naproxen-sulfamethoxazole conjugation showed better anti-inflammatory action by inhibiting induced edema by 82.8%, which is comparable to the medication indomethacin (86.8% inhibition). Whereas it exhibited 75.4% inhibition of COX-2 at 10 µM concentration which is comparable with the reference drug (celecoxib, 77.1% inhibition). Moreover, the binding modes of competitive inhibitors with the urease and COX-2 receptor were predicted through molecular docking studies and their stability analysis through MD simulations showed that these compounds made stable complexes with the respective targets and there were no conformational changes that occurred during simulation. The obtained results showed that the conjugates of approved therapeutic molecules may lead to the development of novel types of pharmacological agents in the treatment of several pathological disorders where urease and COX-2 enzymes are involved.

## 1 Introduction

To cure diseases caused by enzyme disorders, enzyme inhibitors are used. Inhibitors are molecules that can disrupt enzymatic bioactivity by binding themselves to the active site of the enzyme permanently or temporarily. They block the active sites of the enzymes and cease the enzyme-catalyzed biological reaction (Mohiuddin et al., 2019). Enzymes inhibitors are present in nature as well as designed and produced as drugs. Most toxins present in nature are natural enzyme inhibitors. Synthetic enzyme inhibitors find their application in treating diseases while acting as a drug (Kumar et al., 2023; Yakan et al., 2023; Yu et al., 2023). Urease is urea amidohydrolase that catalyzes the hydrolysis of urea into carbon dioxide and ammonia, and it is found in many bacteria, fungi, and plants, as well as in some animals, including humans (Tirmazi et al., 2021; Mehmood et al., 2022). Urease has been studied extensively due to its role in human health, as high levels of urease activity have been linked to various diseases, including urinary tract infections and stomach ulcers. So, the development of effective and secure urease inhibitors has been a significant focus of pharmaceutical studies. Among the many ureases used in enzyme inhibition research, jack bean was the first to be thoroughly described and crystallized (Al-Rooqi et al., 2023). Continuous ammonia production increases the permeability of the gastric mucosa, which leads to inflammation, ulcers, adenocarcinoma, and lymphoma (Ahmed et al., 2020; Imran et al., 2020). Targeting urease activity can help eradicate *H. pylori* (*Helicobacter pylori*) in its early stages of infection because the bacterium depends on it for survival in the stomach’s low pH environment. Our primary research interest is in the design and production of novel urease inhibitors because urease is linked to bacterial infections and there are very few urease inhibitors currently available (Ahmed et al., 2017; Seraj et al., 2021).

An immune system’s protective reaction to injury or infection is inflammation. It is a pathological condition that causes discomfort, stiffness, redness, swelling, and/or tingling (Kaur et al., 2023). Nonsteroidal anti-inflammatory drugs (NSAIDs) are a class of drugs that are widely used to treat pain, fever, and inflammation. The cyclooxygenase (COX) enzymes, which have COX-1 and COX-2 subtypes, are inhibited by NSAIDs. These enzymes are in charge of producing prostaglandins and thromboxanes. COX-1 is present in many tissues throughout the body and is involved in the normal functioning of the stomach, kidneys, and blood platelets. COX-2, on the other hand, is primarily found in cells that are involved in inflammation, such as immune cells and damaged tissues, and their inhibition by NSAIDs results in many side effects (Ahmed et al., 2018b; Manju et al., 2018; Ahmed et al., 2019).

**FIGURE 1:**
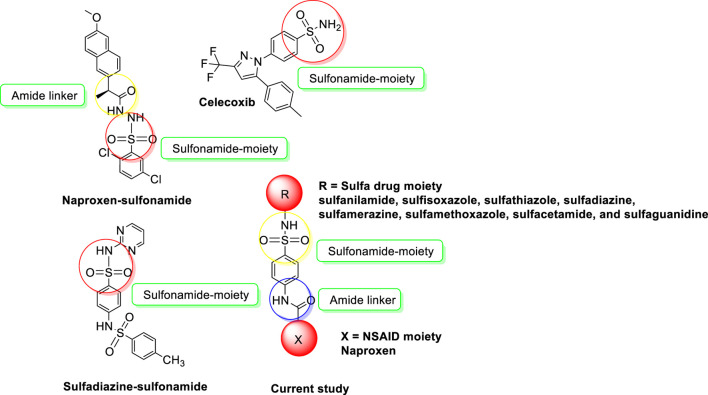
NSAIDs-sulfonamides (current study) and reported molecules as urease inhibitors and anti-inflammatory agents.

Sulfonamides also called sulfa drugs having -SO_2_NH- moiety is derived from the sulfonic acid group (RSO_3_H) by its reaction with the amino group which replaces its hydroxyl group forming sulfonamides. As the sulfonyl group is a constituent of different biologically active molecules, sulfonamides show a wide range of biological activities which secure its unique position in the pharmaceutical drug industry as well as in medicinal chemistry (Peerzada et al., 2021; Elbadawi et al., 2022). The various biological activities exhibited by sulfonamides include diuretic, anti-thyroid, hypoglycemic, anti-conversant, anti-bacterial, anti-hypersensitive, protease inhibitors, anti-diabetic, anti-carbonic anhydrase, anti-urease, anti-migraine, anti-fungal, anti-inflammatory, and herbicidal activities (Abdul Qadir et al., 2015a; Abdul Qadir et al., 2015b; Qadir et al., 2015; Ahmed et al., 2018a; Nadeem et al., 2020; Shahzad et al., 2020). To develop potent and safe urease inhibitors, numerous sulfonamide derivatives, including those of already-marketed drugs, have undergone extensive research in recent years (Figure 1). The results of this study revealed several drug-based compounds that can be used as leading candidates for the continued development of innovative, highly effective urease inhibitors (Seraj et al., 2021; Khan et al., 2022). Sulfonamide derivatives are COX-2 selective inhibitors with fewer side effects that inhibit the COX-2 enzyme responsible for pain and inflammation. Some COX-2-specific enzyme inhibitor sulfonamides are valdecoxib, celecoxib, and parecoxib. These inhibitors have high selectivity and non-steroidal anti-inflammatory therapeutic agents used in the treatment of various diseases. Numerous sulfonamide-tethered NSAIDs with clinical approval have demonstrated encouraging COX-2 inhibitory efficacy, including Celecoxib (Figure 1) (Maier et al., 2004).

Making use of this multi-target approach that is gaining interest among pharmaceutical chemists globally, a series of naproxen-sulfa drug conjugates was designed and synthesized. Synthesis of derivatives has been an important tool and is aimed at modifying the action of existing drugs, particularly to reduce the side effects and to potentiate the action. It is known from the literature more than 60% of the existing drugs are derivatives of the known molecule. Also the drug-drug coupling is a relatively promising approach for new therapeutic targets with a lot of potential (Channar et al., 2017). This approach might overcome the limitations of traditional drug development, and represents an innovative strategy in the field of drug development and has the potential to revolutionize treatment options for various diseases and conditions. The drug-drug coupling for new therapeutic targets based on the ability to target unexplored pathways justify the novelty of this approach and its potential for future drug development in the field of medicinal chemistry.

So in this context, new conjugates were developed by coupling an NSAID (naproxen) and sulfa drugs (sulfanilamide, sulfisoxazole, sulfathiazole, sulfadiazine, sulfamerazine, sulfamethoxazole, sulfacetamide, and sulfaguanidine) through an amide linker as shown in Figure 1. The newly developed conjugates were screened for anti-inflammatory, urease, and COX-2 inhibition. Our design strategy is built on retaining the pharmacophoric moiety in our target molecules. Furthermore, *in silico* studies were performed to check the role of most active inhibitors as ligands against the urease and COX-2 enzymes by molecular docking studies. The stability of most active inhibitors with the said enzymes was also confirmed by analyzing the MD trajectories generated by 50 ns simulation.

## 2 Experimental

### 2.1 Chemistry

#### 2.1.1 General

Newly formulated conjugates were synthesized by utilizing high-purity sulfa drugs originating in Sigma Aldrich, United States, and were purchased from Falcon Scientific, Lahore, Pakistan. NSAIDs were kindly gifted by Novamed Pharmaceuticals, Lahore-Pakistan. Conjugate structures were elucidated through spectral investigations using techniques including FTIR, ^1^HNMR-500 MHz, and ^13^CNMR-125 MHz (Bruker, United States). Thermo Scientific, UK’s HT + elemental analyzer was used for the analysis of the elements (C, H, N, and S). While pre-coated silica TLC plates (Merck, Germany) were used to check the purity of the synthesized conjugates under UV light whereas the Gallenkamp apparatus was used to find out the melting point and are uncorrected.

#### 2.1.2 Synthesis protocol for new conjugates

In a 100 mL flask, naproxen (1 mmol) was dissolved in a solvent containing the mixture of methanol and acetonitrile (30 mL) in a 50:50 ratio. Then 1 mmol of N, N′-Dicyclohexylcarbodiimide (DCC) was added to the solution and the reaction proceeded with the addition of 4-dimethylaminopyridine (DMAP) as a catalyst. The reaction of naproxen and DCC is continued for 30 min at 80°C. Then for amide bond formation, a respective sulfa drug such as sulfanilamide, sulfisoxazole, sulfathiazole, sulfadiazine, sulfamerazine, sulfamethoxazole, sulfacetamide, and sulfaguanidine (1 mmol) was added in the reaction mixture. The refluxing of the reaction mixture was continued for 42 h to complete the reaction, after amide bond formation, dicyclohexylurea (DCU) become precipitated. The TLC was run to monitor the progress of the reaction using ethyl acetate: methanol: n-hexane: DCM (24: 10: 50: 15) as eluent. From the reaction mixture, insoluble DCU was filtered, and the filtrate was separated. The solid product was obtained by evaporating the solvent in rotary evaporated and further purification was done by flash chromatography using acetonitrile/MeOH (25:1) as eluent.

##### 2.1.2.1 (S)-2-(6-methoxynaphthalen-2-yl)-N-(4-sulfamoylphenyl)propanamide (3)

White crystalline solid; yield (%): 76.9; m.p. (^°^C): 208–210; R_f_: 0.74; IR (ATR, υ cm^-1^): 3,463 (sulfonyl-NH), 3,011 (aromatic, =C-H), 2,921 (amide-NH), 2,836 (O-CH_3_), 1710 (-C=O), 1,366 (asymmetric, -NH-S=O), 1,141 (symmetric, -NH-S=O), 1,027 (-S=O); ^1^H NMR (400 MHz, DMSO-d_6_): δ_H_ 8.30 (brs, 1H, NH), 7.74–7.62 (m, 2H, ArH), 7.43 (d, 1H, J = 12.0 Hz, ArH), 7.43 (d, 2H, J = 8.0 Hz, ArH), 7.34 (d, 1H, J = 8.0 Hz, ArH), 7.16–7.12 (m, 1H, ArH), 7.09 (app dt, 1H, J = 8.0, 4.0 Hz, ArH), 6.58 (d, 2H, J = 8.0 Hz, ArH), 5.82 (brs, 2H, NH), 4.01 (q, 1H, J = 8.0 Hz, CH), 3.87 (s, 3H, OCH_3_), 1.45 (d, 3H, J = 4.0 Hz, CH_3_); ^13^C NMR (100 MHz, DMSO- d_6_): δ_C_ 172.8, 160.6, 156.8, 154.0, 152.5, 152.4, 149.8, 130.5, 128.3, 127.9, 125.6, 112.9, 112.8, 112.3, 106.1, 55.6 (O-CH_3_), 48.0, 24.9 (CH_3_); Anal. Calculated for C_20_H_20_N_2_O_4_S (384.45 g/mol): C, 62.48; H, 5.24; N, 7.29; O, 16.65; S, 8.34; Found: C, 62.48; H, 5.24; N, 7.29; O, 16.65; S, 8.34.

##### 2.1.2.2 (S)-N-(4-(N-(3,4-dimethylisoxazol-5-yl)sulfamoyl)phenyl)-2-(6-methoxynaphthalen-2-yl)propanamide (4)

White crystalline solid; yield (%): 71.7; m.p. (^°^C): 241–243; R_f_: 0.82; IR (ATR, υ cm^-1^): 3,463 (sulfonyl-NH), 3,018 (aromatic, =C-H), 2,924 (amide-NH), 2,839 (O-CH_3_), 1710 (-C=O), 1,368 (asymmetric, -NH-S=O), 1,144 (symmetric, -NH-S=O), 1,026 (-S=O); NMR (400 MHz, DMSO-d_6_) δ_H_ 10.06 (brs, 1H, NH), 8.30 (app d, J = 8.0 Hz 1H, ArH), 7.76 (d, 2H, J = 12.0 Hz, ArH), 7.73 (d, 1H, J = 8.0 Hz, ArH), 7.41 (dd, 1H, J = 8.0, 4.0 Hz, ArH), 7.27 (d, 1H, J = 2.4 Hz, ArH), 7.15 (dd, 1H, J = 8.0, 2.4 Hz, ArH), 6.84 (app brs, 1H, J = 4.0 Hz, ArH), 6.59 (1H, obscured by ArH protons), 6.45 (d, 2H, J = 8.0 Hz, ArH), 4.01 (q, 1H, J = 8.0 Hz, CH), 3.86 (s, 3H, OCH_3_), 2.05 (s, 6H, (CH_3_)_2_), 1.45 (d, 3H, J = 4.0 Hz, CH_3_). ^13^C NMR (100 MHz, DMSO) δ_C_ 169.7, 167.4, 159.2, 150.3, 144.8, 141.0, 138.9, 133.6, 128.8, 127.1, 123.6, 121.0, 118.9, 106.1, 55.6 (O-CH_3_), 44.8, 24.5 (CH_3_), 11.0 (CH_3_), 7.3 (CH_3_); Anal. Calculated for C_25_H_25_N_3_O_5_S (479.55 g/mol): C, 62.62; H, 5.25; N, 8.76; O, 16.68; S, 6.69; Found: C, 62.99; H, 5.48; N, 8.87; O, 16.95; S, 6.31.

##### 2.1.2.3 (S)-2-(6-methoxynaphthalen-2-yl)-N-(4-(N-(thiazol-2-yl)sulfamoyl)phenyl)propanamide (5)

White crystalline solid; yield (%): 74.5; m.p. (^°^C): 184–185; R_*f*_: 0.80; IR (ATR, υ cm^-1^): 3,460 (sulfonyl-NH), 3,020 (aromatic, =C-H), 2,924 (amide-NH), 2,838 (O-CH_3_), 1708 (-C=O), 1,366 (asymmetric, -NH-S=O), 1,141 (symmetric, -NH-S=O), 1,024 (-S=O); ^1^H NMR (400 MHz, DMSO-*d*_6_): 7.82–7.75 (m, 2H, ArH), 7.72 (brs, 1H, NH), 7.66 (s, 1H, ArH), 7.41 (d, 1H, J = 8.0 Hz, ArH), 7.38 (d, 2H, J = 8.0 Hz, ArH), 7.27 (d, 2H, J = 2.4 Hz, ArH), 7.15 (td, 1H, J = 8.0, 2.4 Hz, ArH), 6.91 (d, 1H, J = 8.0 Hz, ArH), 6.46 (d, 1H, J = 8.0 Hz, =CH-N), 6.40 (d, 1H, J = 4.0 Hz, =CH-S), 5.59 (d, 1H, J = 8.0 Hz, ArH), 4.01 (q, 1H, J = 8.0 Hz, CH), 3.87 (s, 3H, OCH_3_), 1.47 (d, 3H, J = 4.0 Hz, CH_3_); ^13^C NMR (100 MHz, DMSO- *d*_6_): δC 167.4, 159.2, 150.3, 144.8, 141.0, 138.9, 133.6, 128.8, 127.1, 123.6, 121.0, 118.9, 106.1, 55.6 (O-CH_3_), 11.0 (CH_3_); Anal. Calculated for C_23_H_21_N_3_O_4_S_2_ (467.56 g/mol): C, 59.08; H, 4.53; N, 8.79; O, 13.69; S, 13.71; Found: C, 59.20; H, 4.67; N, 8.61; O, 13.95; S, 13.41.

##### 2.1.2.4 (S)-2-(6-methoxynaphthalen-2-yl)-N-(4-(N-(pyrimidin-2-yl)sulfamoyl)phenyl)propanamide (6)

White crystalline solid; yield (%): 69.2; m.p. (^°^C): 163–165; R_*f*_: 0.72; IR (ATR, υ cm^-1^): 3,461 (sulfonyl-NH), 3,016 (aromatic, =C-H), 2,922 (amide-NH), 2,840 (O-CH_3_), 1710 (-C=O), 1,368 (asymmetric, -NH-S=O), 1,144 (symmetric, -NH-S=O), 1,026 (-S=O); ^1^H NMR (400 MHz, DMSO-*d*_6_): 8.42 (d, 2H, J = 8.0 Hz, ArH), 7.82 (d, 2H, J = 12.0 Hz, ArH), 7.74 (d, 1H, J = 8.0 Hz, ArH), 7.67 (brs, 1H, NH), 7.60 (d, 1H, J = 8.0 Hz, ArH), 7.41 (dd, 1H, J = 8.0, 4.0 Hz, ArH), 7.27 (d, 1H, J = 2.4 Hz, ArH), 7.15 (dd, 1H, J = 8.0, 2.4 Hz, ArH), 6.92 (t, 1H, J = 4.0 Hz, ArH), 6.59 (dd, 1H, J = 8.0, 4.0 Hz, ArH), 6.54 (d, 2H, J = 8.0 Hz, ArH), 4.01 (q, 1H, J = 8.0 Hz, CH), 3.86 (s, 3H, OCH_3_), 1.45 (d, 3H, J = 4.0 Hz, CH_3_); ^13^C NMR (100 MHz, DMSO- *d*_6_): 171.8, 158.5, 157.5, 154.0, 149.7, 137.1, 133.6, 130.1, 129.6, 128.8, 127.1, 127.0, 126.1, 118.9, 112.5, 107.2, 106.1, 55.6 (O-CH_3_), 43.9, 19.8 (CH_3_); Anal. Calculated for C_24_H_22_N_4_O_4_S (462.52 g/mol): C, 62.32; H, 4.79; N, 12.87; O, 12.11 13.84; S, 6.93; Found: C, 62.49; H, 4.98; N, 12.73; O, 13.95; S, 6.98.

##### 2.1.2.5 (S)-2-(6-methoxynaphthalen-2-yl)-N-(4-(N-(4-methylpyrimidin-2-yl)sulfamoyl)phenyl)propanamide (7)

White crystalline solid; yield (%): 77.6; m.p. (^°^C): 160–162; R_*f*_: 0.71; IR (ATR, υ cm^-1^): 3,460 (sulfonyl-NH), 3,014 (aromatic, =C-H), 2,918 (amide-NH), 2,839 (O-CH_3_), 1710 (-C=O), 1,368 (asymmetric, -NH-S=O), 1,144 (symmetric, -NH-S=O), 1,026 (-S=O); ^1^H NMR (400 MHz, DMSO-*d*_6_): 8.34 (d, 1H, J = 8.0 Hz, ArH), 7.76 (d, 2H, J = 12.0 Hz, ArH), 7.74 (d, 1H, J = 8.0 Hz, ArH), 7.67 (brs, 1H, NH), 7.60 (d, 1H, J = 8.0 Hz, ArH), 7.41 (dd, 1H, J = 8.0, 4.0 Hz, ArH), 7.27 (d, 1H, J = 2.4 Hz, ArH), 7.15 (dd, 1H, J = 8.0, 2.4 Hz, ArH), 6.81 (app d, 1H, J = 4.0 Hz, ArH), 6.59 (dd, 1H, J = 8.0, 4.0 Hz, ArH), 6.54 (d, 2H, J = 8.0 Hz, ArH), 4.01 (q, 1H, J = 8.0 Hz, CH), 3.86 (s, 3H, OCH_3_), 2.29 (s, 3H, CH_3_), 1.45 (d, 3H, J = 4.0 Hz, CH_3_); ^13^C NMR (100 MHz, DMSO- *d*_6_): 171.9, 158.5, 157.5, 154.0, 149.7, 137.1, 133.6, 130.1, 129.6, 128.8, 127.1, 126.1, 118.9, 112.4, 106.1, 55.6 (O-CH_3_), 43.8, 24.9, 19.8 (CH_3_); Anal. Calculated for C_25_H_24_N_4_O_4_S (476.55 g/mol): C, 63.01; H, 5.08; N, 11.76; O, 13.43; S, 6.73; Found: C, 62.99; H, 5.28; N, 11.87; O, 13.95; S, 6.81.

##### 2.1.2.6 (S)-2-(6-methoxynaphthalen-2-yl)-N-(4-(N-(5-methylisoxazol-3-yl)sulfamoyl)phenyl)propanamide (8)

White crystalline solid; yield (%): 75.4; m.p. (^°^C): 178–180; R_*f*_: 0.83; IR (ATR, υ cm^-1^): 3,460 (sulfonyl-NH), 3,014 (aromatic, =C-H), 2,918 (amide-NH), 2,839 (O-CH_3_), 1708 (-C=O), 1,368 (asymmetric, -NH-S=O), 1,142 (symmetric, -NH-S=O), 1,026 (-S=O); ^1^H NMR (400 MHz, DMSO-*d*_6_): 10.06 (brs, 1H, NH), 7.76 (d, 2H, J = 12.0 Hz, ArH), 7.73 (d, 1H, J = 8.0 Hz, ArH), 7.67 (brs, 1H, NH), 7.41 (dd, 1H, J = 8.0, 4.0 Hz, ArH), 7.27 (d, 1H, J = 2.4 Hz, ArH), 7.15 (dd, 1H, J = 8.0, 2.4 Hz, ArH), 6.84 (app brs, 1H, J = 4.0 Hz, ArH), 6.59 (1H, obscured by ArH protons), 6.45 (d, 2H, J = 8.0 Hz, ArH), 4.01 (q, 1H, J = 8.0 Hz, CH), 3.86 (s, 3H, OCH_3_), 2.05 (s, 3H, CH_3_), 1.45 (d, 3H, J = 4.0 Hz, CH_3_); ^13^C NMR (100 MHz, DMSO- *d*_6_): δC 171.9, 157.7, 154.4, 149.7, 136.2, 133.8, 129.6, 128.9, 127.5, 126.7, 126.1, 119.3, 112.8, 107.2, 106.2, 55.6 (O-CH_3_), 44.8, 18.9 (CH_3_), 12.6 (CH_3_); Anal. Calculated for C_24_H_23_N_3_O_5_S (465.52 g/mol): C, 61.92; H, 4.98; N, 9.03; O, 17.18; S, 6.89; Found: C, 61.88; H, 4.91; N, 9.17; O, 17.25; S, 6.95.

##### 2.1.2.7 (S)-N-(4-(N-acetylsulfamoyl)phenyl)-2-(6-methoxynaphthalen-2-yl)propanamide (9)

White crystalline solid; yield (%): 73.4; m.p. (^°^C): 138–140; R_*f*_: 0.73; IR (ATR, υ cm^-1^): 3,462 (sulfonyl-NH), 3,014 (aromatic, =C-H), 2,918 (amide-NH), 2,839 (O-CH_3_), 1710 (-C=O), 1,368 (asymmetric, -NH-S=O), 1,144 (symmetric, -NH-S=O), 1,028 (-S=O); ^1^H NMR (400 MHz, DMSO-*d*_6_): 7.80–7.75 (m, 1H, ArH), 7.71 (brs, 1H, NH), 7.65 (s, 1H, ArH), 7.43 (app dd, 1H, J = 8.0, 4.0 Hz, ArH), 7.38 (d, 2H, J = 8.0 Hz, ArH), 7.27 (d, 2H, J = 2.4 Hz, ArH), 7.14 (app dd, 1H, J = 8.0, 2.4 Hz, ArH), 7.01 (d, 1H, J = 4.0 Hz, ArH), 6.58 (d, 1H, J = 8.0 Hz, ArH), 4.01 (q, 1H, J = 8.0 Hz, CH), 3.87 (s, 3H, OCH_3_), 1.50 (s, 3H, CH_3_), 1.47 (d, 3H, J = 4.0 Hz, CH_3_); ^13^C NMR (100 MHz, DMSO- *d*_6_): 175.6, 160.6, 156.8, 154.0, 152.5, 152.4, 149.8, 133.6, 130.5, 128.3, 125.6, 112.9, 112.9, 112.3, 106.1, 56.8 (O-CH_3_), 47.9, 33.8, 27.3 (CH_3_); Anal. Calculated for C_22_H_22_N_2_O_5_S (426.49 g/mol): C, 61.96; H, 5.2; N, 6.57; O, 18.76; S, 7.52; Found: C, 61.78; H, 5.35; N, 6.87; O, 18.95; S, 7.31.

##### 2.1.2.8 (S)-N-(4-(N-carbamimidoylsulfamoyl)phenyl)-2-(6-methoxynaphthalen-2-yl)propanamide (10)

White crystalline solid; yield (%): 68.7; m.p. (^°^C): 202–204; R_*f*_: 0.76; IR (ATR, υ cm^-1^): 3,464 (sulfonyl-NH), 3,014 (aromatic, =C-H), 2,921 (amide-NH), 2,839 (O-CH_3_), 1710 (-C=O), 1,368 (asymmetric, -NH-S=O), 1,144 (symmetric, -NH-S=O), 1,026 (-S=O); ^1^H NMR (400 MHz, DMSO-*d*_6_): 7.82–7.75 (m, 1H, ArH), 7.72 (brs, 1H, NH), 7.66 (s, 1H, ArH), 7.41 (d, 1H, J = 8.0 Hz, ArH), 7.38 (d, 2H, J = 8.0 Hz, ArH), 7.27 (d, 2H, J = 8.0, 2.4 Hz, ArH), 7.15 (td, 1H, J = 8.0, 2.4 Hz, ArH), 6.91 (d, 1H, J = 8.0 Hz, ArH), 6.46 (d, 1H, J = 8.0 Hz, ArH), 6.40 (t, 1H, J = 8.0 Hz, NH), 5.59 (d, 2H, J = 8.0 Hz, NH_2_), 4.01 (q, 1H, J = 8.0 Hz, CH), 3.87 (s, 3H, OCH_3_), 1.47 (d, 3H, J = 4.0 Hz, CH_3_); ^13^C NMR (100 MHz, DMSO- *d*_6_): δC 160.2, 157.8, 152.0, 144.8, 141.0, 138.9, 133.6, 130.5, 128.3, 127.9, 125.6, 112.9, 112.9, 112.3, 106.1, 59.6 (O-CH_3_), 48.7, 25.7 (CH_3_); Anal. Calculated for C_20_H_20_N_2_O_4_S (426.49 g/mol): C, 59.14; H, 5.2; N, 13.14; O, 15.01; S, 7.52; Found: C, 59.23; H, 5.38; N, 13.45; O, 16.25; S, 7.31.

### 2.2 Pharmacological activities

#### 2.2.1 Antiurease assay

With a few minor modifications, the urease inhibition experiment was carried out as described in our past research (Ahmed et al., 2017; Ahmed et al., 2020; Imran et al., 2020). Briefly, DMSO was used to dissolve the synthesized conjugates (inhibitors, 250–0.49 µM) and reference urease inhibitor (thiourea). Each falcon tube contains the respective inhibitor (20 µL), a buffer of pH = 6.8–7.0 (K_2_HPO_4_, 100 μL, 50 mM), and jack bean urease (20 µL), each tube was mixed well, and the mixture was incubated at 37°C for 30 min. Each tube received 400 µL of urea (20 mM) as substrate, which was then incubated for 10 min at the same temperature. Afterward, each tube received 400 µL of phenol reagent and 750 µL of alkali reagent containing 0.1% active chlorine and was left at 37°C for 50 min. Following the use of a spectrophotometer (Labdex, LX210DS, United Kingdom) to measure the absorbance of the mixture in each tube at 595 nm, the percentage of urease inhibition was calculated using the equation below.

$$\%\mathrm{Urease}\;\mathrm{inhibition}=\left(1-T/C\right)\times 100$$ (1)

Where T and C are the absorbances of each well-containing inhibitor and blank respectively, each assay was performed in triplicate, and results are presented as mean ± SEM. Using a regression equation where 50% inhibition was seen, the IC_50_ values of each inhibitor were determined. Each inhibitor’s binding mechanism was tested at various doses (0–20 µM) for kinetics investigations. Urea was used as substrate in different concentrations (0.5–4.0 mM) to determine the mode of inhibition of inhibitors whether these acted as uncompetitive, mixed (non-competitive), or competitive. Lineweaver Burk plots were drawn using GraphPad PRISM 7.0 to determine the values of K_m (app)_, V_max (app)_, and K_i_ (inhibition constant).

#### 2.2.2 Anti-inflammatory studies

As previously mentioned in our study, carrageenan-induced acute inflammation in mice was used to measure the anti-inflammatory effect (Ahmed et al., 2018b). Six groups of mice were formed and each group comprised six mice, the mice fasted 16 h before of induction of inflammation by injecting the phlogistic agent (carrageenan). The right hind paw of each mouse was measured before and after the 100 µL injection of the carrageenan (1% in 0.9% saline). The test substances, standard medication (indomethacin), and control (0.9% saline) were administered intraperitoneally at 10 mg/kg body weight. For 3 h, and after the 1-h interval, the thickness (mm) of the right hind paw of each mouse was measured. The difference in thickness of paw edema of control and test substance was used to determine the percentage inhibition of inflammation by the formula given below.

$$\mathrm{Percentage inhibition of inflammation}=\frac{{\left(C_{t}-C_{o}\right)}_{\mathrm{control}}{\left(C_{t}-C_{o}\right)}_{\mathrm{treated}}}{{\left(C_{t}-C_{o}\right)}_{\mathrm{control}}}\times 100$$ (2)

Where C_t_ and C_o_ are the right hind paw thickness after and before carrageenan injection.

#### 2.2.3 COX-2 inhibitory assay

The inhibition of COX-2 was performed by using a kit procured from Cayman Chemical Company, United States (Item No. 760151). For the initial activity assay, 150 µL buffer, 10 µL heme, and 10 µL COX-2 enzyme were mixed in a well whereas 110 µL buffer, 10 µL heme, 10 µL COX-2 enzyme, and 40 µL test inhibitor (10 µM) were mixed for inhibitory assay using 96 wells plate. The well plate was placed in a shaker for 5 min at 25°C, then each well received the colorimetric substrate (20 µL), and arachidonic acid (20 µL) was added to initiate the reaction. Then the well plate was incubated at 25°C for 5 min, following the use of a microplate reader (Labtech, LT-4500, United Kingdom) to measure the absorbance of the mixture in each well at 590 nm, the percentage of COX-2 inhibition was calculated using the equation below (Ahmed et al., 2018b), each assay was performed in triplicate.

$$\%\mathrm{COX}-2\;\mathrm{inhibition}=1-\frac{T}{C}\times 100$$ (3)

Where T and C are the absorbances of each well-containing inhibitor and blank.

### 2.3 Molecular docking and dynamics simulation studies

The binding modes of competing inhibitors in the urease and COX-2 pockets were predicted using molecular docking. To achieve this, the crystallographic structures of urease (PDB ID: 4H9M) and COX-2 (PDB ID: 3NTG) were prepared for docking using the Maestro (Schrödinger, 2017). The processing of the receptors—which included the inclusion of hydrogen atoms, the appointing of bond orders, and the production of zero bond orders for metals—was done to maintain their structural integrity. Additionally, unnecessary protein chains and water molecules were removed. The tautomeric states of the structures were adjusted to refine the structure along with protonation at pH 7.4. The geometries of the structures were optimized by hydrogen bond assignment at neutral pH and then minimized using the OPLS_2005 forcefield (Shivakumar et al., 2012). Subsequently, site-specific grids were generated to select the co-crystal ligands in the respective receptors. The competitive conjugates were also prepared using the LigPrep tool, and the lowest energy conformers of the conjugates were obtained for the molecular docking analysis.

The best binding poses of the conjugates complexed with urease and COX-2 were subjected to 50ns using VMD (Humphrey et al., 1996) and NAMD (Phillips et al., 2020) to explore their stability. As starting point, the initial files required to run the simulation were prepared using the modules of Ambertools 21 (Case et al., 2021). The antechamber modules were used to generate the parameters of conjugates while Leap Program was used to add the missing hydrogen atoms in the protein structures (Case et al., 2005). After parameterization, TIP3P water molecules were added to the systems in a periodic box of 10 Å (Jorgensen and Chandrasekhar, 1983) and then these were neutralized by the addition of Na^+^ ions. The energy clashes were removed by minimizing the system by using ff14SB forcefield (Duan et al., 2003) for protein and GAFF for ligands. After minimization, the solvation was equilibrated for 10,000 steps which were followed by the temperature equilibrations at 200, 250, and 300 K. The final equilibrated systems was then subjected to a 50 ns production run and the trajectories were stored at every 2 ps for the analysis. The analysis of the MD trajectories was conducted by using the BIO3D package of R (Grant et al., 2021).

## 3 Results and discussion

### 3.1 Chemistry

**SCHEME 1:**
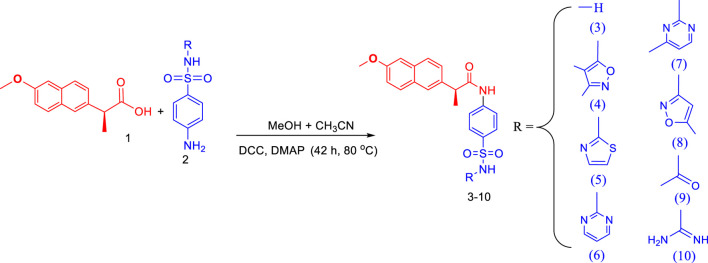
Synthesis procedure for naproxen-sulfa drugs conjugates.

The synthesis of target conjugates (Scheme 1) was performed by coupling the naproxen with sulfa drugs (sulfanilamide, sulfisoxazole, sulfathiazole, sulfadiazine, sulfamerazine, sulfamethoxazole, sulfacetamide, and sulfaguanidine). For coupling of amino groups of sulfa drugs with the hydroxyl of acetylsalicylic acid, DCC was used as a coupling agent in the presence of DMAP as a catalyst. Several investigations have revealed that the most widely employed reaction in medicinal chemistry is, in fact, amide coupling. The synthesis of a huge variety of compounds is possible due to a well-known reaction between two readily available synthons, a carboxylic acid, and an amine. In recent years, sulfonamide linkers have become more widely used in medicinal chemistry (Ertl et al., 2023). The details of synthesis are presented in the experimental section. The characterization of the newly synthesized conjugates was done by various spectroscopic techniques like ^1^H-NMR, ^13^C-NMR, IR, and elemental analysis, and details are shown in Section 2.1.2.

In the IR spectrum of compounds, the -NH moiety present in sulfonamides showed the absorption band at 3,460–3,464 cm^−1^. The absorption bands at 3,011–3,020 cm^−1^ represent the -NH of acetamide moiety in compounds. The -NH-S=O group showed the absorption bands in 1,366–1,368 cm^−1^ (unsymmetrical), and 1,141–1,144 cm^−1^ (symmetrical) regions. In IR spectra of all newly synthesized compounds, the presence of the -S=O group is confirmed by the absorption band appearing in the region 1,024–1,028 cm^−1^. In proton NMR (^1^HMR) spectra of conjugates, the peaks appearing at δ 7.67–7.76 ppm exhibit the presence of–NH proton of the sulfonamide group confirming the–SO_2_NH- group. The values of chemical shifts and integrals of all remaining aromatic and aliphatic protons are already mentioned in the experimental section of 2.1.2. The peaks at 167.4–174.9 ppm in ^13^C NMR spectra show the presence of a carbonyl carbon group present in the compounds. The spectral ^13^C NMR analysis of all compounds with assigned structures was consistent.

### 3.2 Pharmacological activities

#### 3.2.1 Urease inhibition and structure activity relationship (SAR)

We evaluated the conjugates that had been successfully synthesized for their *in vitro* anti-urease action. In urease inhibition investigations, thiourea has served as a reference and exhibited the IC_50_ value of 22.61 *µ*M. Table 1 presented the enzyme (urease) inhibition data, all of the conjugates are effective against it.

**TABLE 1:** IC_50_ and kinetics parameters of naproxen-sulfa drugs conjugates (**3–10**).

Conjugate	IC_50_ (µM); mean ± SEM	^a^ V_max (app)_	^b^ K_m (app)_	^c^ K_i_ (µM)	Mode of inhibition
	(% inhibition), n = 3^e^	(µM/min)	(mM)		
**3**	6.69 ± 0.11 (89.4)	2.57	8.33	2.40	Competitive
**4**	25.63 ± 0.24 (94.1)	-	-	-	-
**5**	5.82 ± 0.28 (88.9)	0.714	3.03	5.05	Competitive
**6**	4.08 ± 0.10 (90.3)	0.363	1.58	5.58	Mixed
**7**	16.57 ± 0.14 (84.3)	0.602	2.96	9.98	Mixed
**8**	29.64 ± 0.27 (87.6)	-	-	-	-
**9**	20.32 ± 0.12 (85.7)	0.66	1.14	2.61	Mixed
**10**	5.06 ± 0.29 (89.1)	1.96	4.32	3.56	Competitive
^d^ Thiourea	22.61 ± 0.23 (92.3)	18.61	2.18	18.18	Competitive

^a^ V_max (app)_ = Intercept, the maximum rate at 20 µM of inhibitor concentration.

^b^ K_m (app)_ = Slope of the line (Michaelis constant) at 20 µM of inhibitor concentration.

^c^ K_i (_µM) = Inhibition constant.

^d^ Standard urease inhibitor.

^e^ Each assay was performed in triplicate.

**SCHEME 2:**
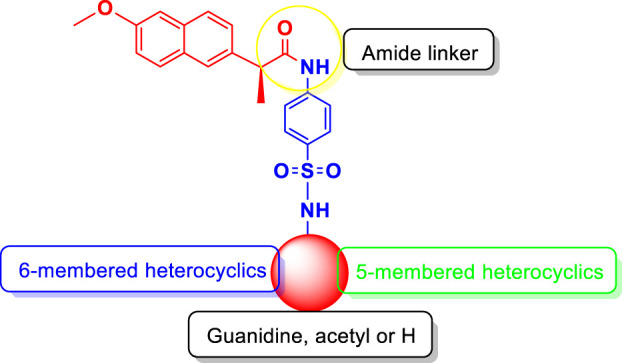
Illustration of SAR studies.

SAR studies (Scheme 2) were carried out purely based on the central core containing naproxen moiety coupled with various substituted sulfonamides (sulfa drugs) through an amide linker. The effective structural feature of the most active inhibitor comprised of five membered heterocyclics such as thiazole, and isoxazole substituted sulfonamide. Naproxen coupled to thiazole substituted sulfonamides (sulfathiazole) demonstrated the five time more activity (**5**, IC_50_ = 5.82 ± 0.28 µM) than the methylisoxazole (**8**, IC_50_ = 29.64 ± 0.27 µM, sulfamethoxazole) and dimethylisoxazole (**4**, IC_50_ = 25.63 ± 0.24 µM, sulfafurazole/sulfisoxazole) substitutes sulfa drugs. It is evident that the dimethylisoxazole is more active than the mono-methylisoxazole which is due to electron donating effect of methyl group. In contrast, the pyrimidine (**6**, IC_50_ = 4.08 ± 0.10) and methylpyrimidin (**7**, IC_50_ = 16.57 ± 0.14) had shown more activity than isoxazole substituted sulfonamides (**4** and **8**). Furthermore, guanidine (**10**, IC_50_ = 5.06 ± 0.29), and amino (**3**, IC_50_ = 6.69 ± 0.11) groups on sulfonamide side showed the excellent urease inhibition activities as compared to five and six membered heterocyclic substituents (Table 1). Whereas acetyl (**9**, IC_50_ = 20.32 ± 0.12) substitution on sulfonamide side demonstrated less urease inhibition than the guanidine and amino substituent but more active than methylisoxazole and dimethylisoxazole on sulfonamide side.

#### 3.2.2 Enzyme kinetic studies

**FIGURE 2:**
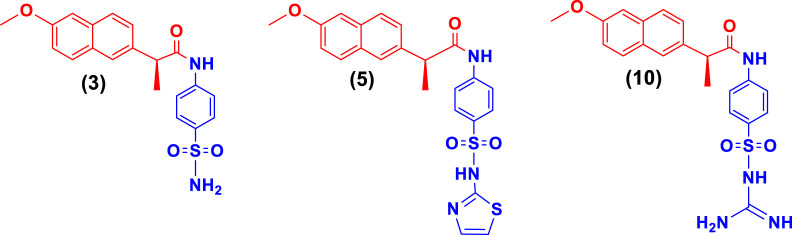
Illustration of competitive mode of urease inhibitors.

Naproxen conjugated (Figure 2) with sulfanilamide (**3**), sulfathiazole (**5**), and sulfaguanidine (**10**) was found potent and showed a **competitive** mode of urease inhibition, with IC_50_ (µM) values 6.69 ± 0.11, 5.82 ± 0.28, 5.06 ± 0.29, and urease inhibition was 89.4%, 88.9%, and 89.1% respectively (Table 1).

The competitive mode of inhibition of conjugates (**3, 5, and 10**) was demonstrated by kinetic studies. The kinetics studies were performed by using five different concentrations (0.0, 5.0, 10.0, 15.0, and 20.0 µM) of each conjugate while using four different conditions of urea (0.5, 1.0, 2.0, and 4.0) as substrate.

**FIGURE 3:**
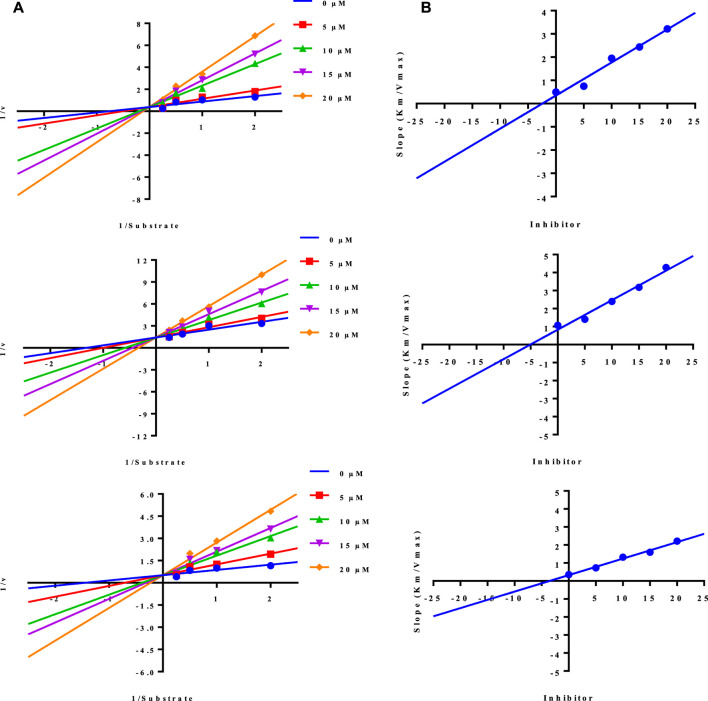
Demonstration of the competitive mode of inhibition of conjugates (3, 5, and 10) by kinetic studies **(A)** Primary Lineweaver Burk plot for K_m_, and V_max_ values calculation, **(B)** Secondary Lineweaver Burk plot for K_i_ value calculation.

Lineweaver-Burk plot is a powerful tool for analyzing enzyme kinetics and determining the mode of inhibition of an enzyme by a particular conjugate. The inhibitor molecule (conjugate) binds to the enzyme’s active site in competitive inhibition and prevents the substrate from attaching. In a Lineweaver-Burk plot, competitive inhibition is characterized by a change in the slope of the line (K_m_, also called the Michaelis constant), while the intercept remains the same (V_max_, the maximum rate). The increase in the K_m_ value of the urease enzyme while the value of V_max_ remains constant at 20 µM of inhibitor concentration demonstrated that the conjugates (**3, 5, and 10**) inhibit the enzyme in a competitive way (Figure 3).

The inhibition constant (K_i_) value of each conjugate was also calculated by plotting the slope of each line vs. different concentrations of each conjugate, also called secondary Lineweaver Burk secondary plots. The K_i_ value of conjugates (**3, 5, and 10**) was found 2.40, 5.05, and 3.56 µM respectively (Table 1). The plots of enzymatic kinetics of competitive inhibitors are presented below in Figure 3.

**FIGURE 4:**
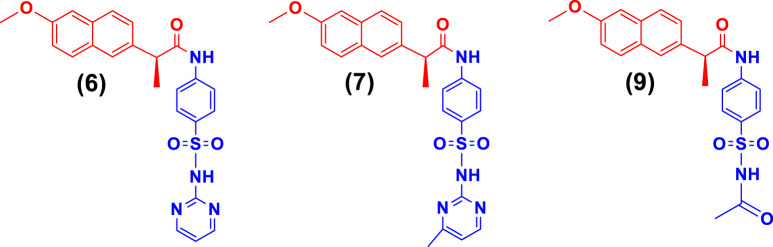
Illustration of mixed mode of urease inhibitors.

While the rest of the conjugates also showed good inhibition for the urease in the range between 84.3% and 94.1% and IC_50_ values ranged between 4.08 ± 0.10 and 29.64 ± 0.27 µM. Naproxen conjugated with sulfadiazine (**6**), sulfamerazine (**7**), and sulfacetamide (**9**) exhibited **a mixed** mode of urease inhibition (Figure 4).

When there is a mixed type of inhibition, the inhibitor molecule binds to the enzyme-substrate complex, preventing the reaction from occurring. By boosting the concentration of the substrate, this form of inhibition cannot be overcome. In a Lineweaver-Burk plot, mixed-type inhibition is characterized by a change in both the slope (K_m_) and the intercept of the line (V_max_).

**FIGURE 5:**
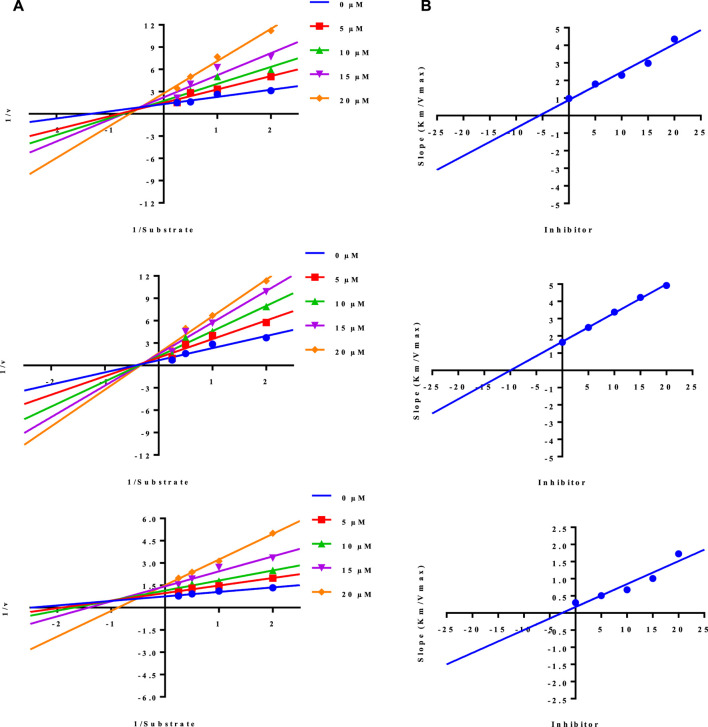
Demonstration of the mixed mode of inhibition of conjugates (6, 7, and 9) by kinetic studies **(A)** Primary Lineweaver Burk plot for K_m_, and V_max_ values calculation, **(B)** Secondary Lineweaver Burk plot for K_i_ value calculation.

The mixed type of inhibition of conjugates (**6, 7, and 9**) were also demonstrated after kinetics studies by using five different concentration (0.0, 5.0, 10.0, 15.0, and 20.0 µM) of each conjugate while using four different conditions of urea (0.5, 1.0, 2.0, and 4.0) as substrate. The increase in K_m_ value of the urease enzyme while the value of V_max_ decreases at 20 µM of inhibitor concentration demonstrated that the conjugates (**6, 7, and 9**) inhibit the enzyme inhibition in a mixed way (Figure 5). The K_i_ values calculated from secondary Lineweaver Burk secondary plots and were obtained 5.58, 9.98, and 2.61 µM (Table 1) for conjugates (**6, 7 and 9**) respectively. The plots of enzymatic kinetics of mixed inhibitors are presented below in Figure 3.

#### 3.2.3 Anti-inflammatory and COX-2 inhibition studies

Naproxen conjugated containing isoxazole and thiazole moieties (**4, 5, and 8**) were evaluated for their potential anti-inflammatory action whereas indomethacin was used as a reference NSAID (Table 2). Heterocyclic scaffolds have a wide variety of structural variations and have been shown to simultaneously target several inflammatory pathways. Numerous mediators and signaling channels interact intricately during inflammation. Contrary to medications that predominantly target a specific enzyme or pathway, heterocyclics have the potential to affect various elements of the inflammatory cascade, offering a wider spectrum of anti-inflammatory efficacy. In this context, the conjugates (**4**, **5**, and **8**) contain the five-membered heterocyclics, and their selection as anti-inflammatory agents was hypothesized as celecoxib contains the five-membered heterocyclic. The inhibitory activities of these conjugates were also assessed *in vitro* against both COX-2 and celecoxib was used as the standard inhibitor of COX-2. COX-2 enzyme screening kit was used to evaluate the inhibition of enzyme and the % inhibition of COX-2 enzyme by each conjugate under study is presented in Table 2.

**TABLE 2:** Anti-inflammatory and COX-2 inhibition studies of naproxen-sulfa drugs conjugates.

Treatment	Paw edema in mm, (mean ± SEM), n = 6^a^, (% inhibition)				% inhibition (n = 3)** COX-2 at 10 µM
	0 h	1 h	2 h	3 h	
**4**	1.80 ± 0.04 (−)	2.72 ± 0.12 (8.2)	2.53 ± 0.09 (45.5)	2.36 ± 0.11 (78.0)	64.5
**5**	1.81 ± 0.05 (−)	2.71 ± 0.09 (9.1)	2.57 ± 0.11 (43.3)	2.43 ± 0.08 (75.3)	61.3
**8**	1.80 ± 0.08 (−)	2.56 ± 0.14 (24.1)	2.51 ± 0.08 (47.0)	2.23 ± 0.12 (82.8)	75.4
Naproxen	1.81 ± 0.06 (−)	2.60 ± 0.13 (21.1)	2.53 ± 0.11 (46.3)	2.41 ± 0.12 (76.1)	-
Standard drugs^b^ ^,^ ^c^	1.81 ± 0.05 (−)	2.61 ± 0.14 (20.1)	2.41 ± 0.09 (55.2)	2.14 ± 0.09 (86.8)	77.1
Control^d^	1.80 ± 0.04 (−)	2.81 ± 0.11 (−)	3.14 ± 0.12 (−)	4.31 ± 0.11 (−)	-

^a^ For paw edema, each group comprise of six mice, ** each assay was performed in triplicate.

^b^ Indomethacin = Reference NSAID.

^c^ Celecoxib = Reference COX-2, inhibitor.

^d^ Saline = 0.9%.

Using carrageenan-induced paw edema, the anti-inflammatory efficacy of synthetic conjugates was evaluated in the current study. When carrageenan is injected into the paw of an animal, it induces a local inflammatory response that results in swelling (edema). This response involves the release of pro-inflammatory mediators such as prostaglandins, leukotrienes, and cytokines. The biphasic nature of carrageenan-induced edema makes it a useful model for studying the mechanisms of acute inflammation and evaluating the anti-inflammatory activities of drugs and natural compounds. Broad-spectrum anti-inflammatory compounds exhibit action against both the early and late stages of carrageenan-induced edema (Rathod et al., 2023).

Histamine, serotonin, and bradykinin are released during the early stages of carrageenan-induced edema, which starts within the first few hours of carrageenan administration. Usually lasting 1–2 h, this phase is characterized by increased vascular permeability, vasodilation, and neutrophil infiltration. The second phase of carrageenan-induced edema occurs approximately 2–3 h after carrageenan injection and is characterized by the release of prostaglandins and other mediators of inflammation. This phase is more sustained than the first phase, typically lasting for several hours, and is associated with increased vascular permeability, leukocyte infiltration, and tissue damage (de Siqueira Patriota et al., 2022). Table 2, presented the anti-inflammatory data which revealed that the conjugates significantly inhibited the induced edema in the late phase.

Naproxen-sulfamethoxazole conjugate (**8**), among the tested conjugates, demonstrated better anti-inflammatory action by inhibiting 82.8% of induced edema, and the inhibition result is comparable to the indomethacin (86.8%) which was used as reference anti-inflammatory drug. For the COX-2 inhibition studies, the results demonstrated that conjugate 8 also exhibited 75.4% inhibition which is comparable with the reference drug (celecoxib, 77.1% inhibition). Naproxen conjugated with sulfamethoxazole exhibited better COX-2 inhibition than thiazole-conjugated moiety.

### 3.3 Molecular docking and dynamics simulation studies

**FIGURE 6:**
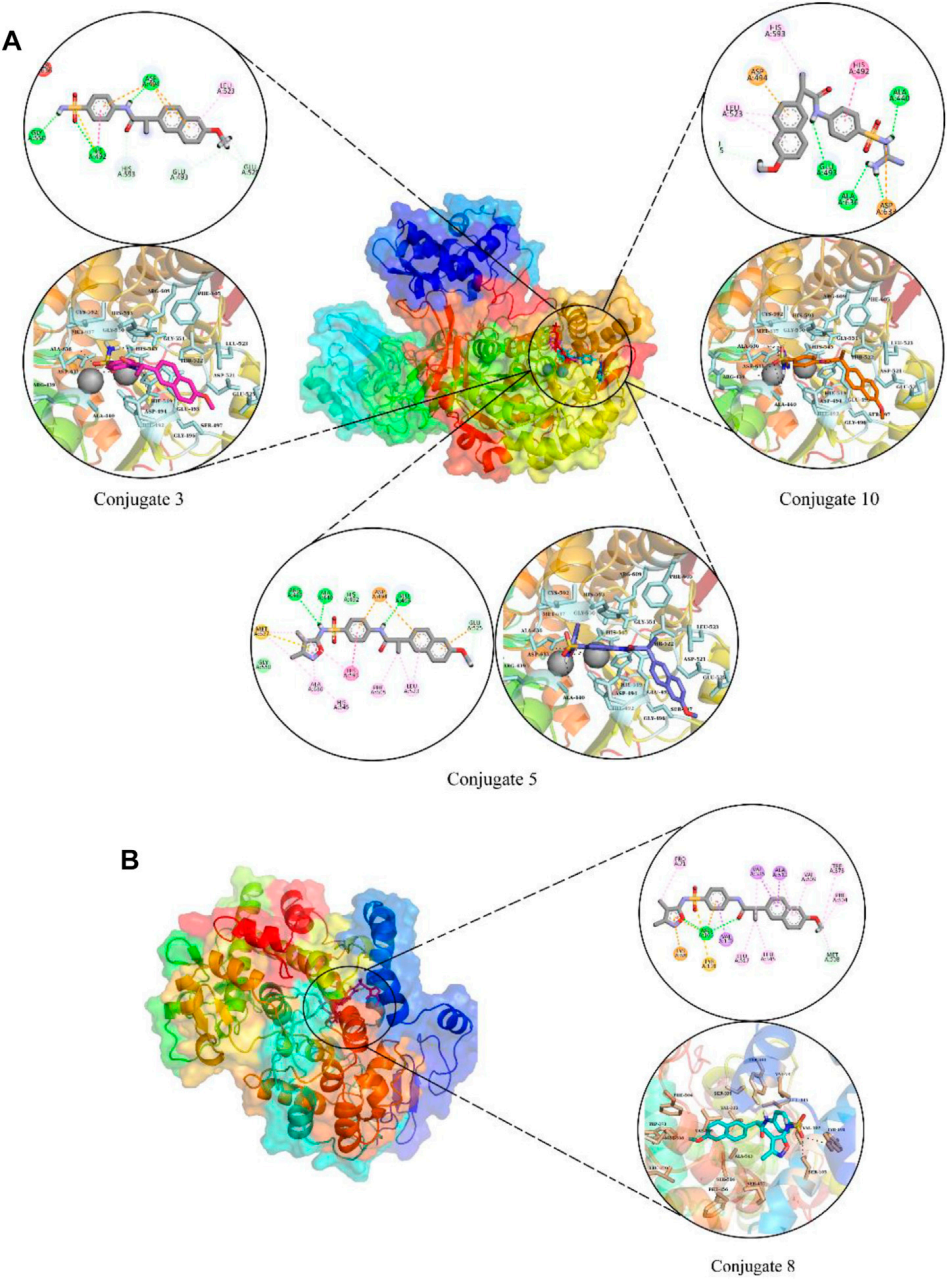
The molecular interactions and plausible binding modes of competitive inhibitors with urease **(A)** and COX-2 **(B)**. In molecular interactions, green spheres show hydrogen bonds, orange show pi-sulfur interactions and magenta spheres show hydrophobic interactions.

Molecular docking was used to estimate the likely binding modalities of the competitive inhibitors, and the molecular interactions were examined. In urease docking studies, it was observed that conjugate **3** made three hydrogen bonds with His492, Asp494, and Gly550, one pi-pi interaction with His492, and one hydrophobic interaction with Leu523. Conjugate **5** was involved in hydrogen bonding with Arg439, Ala440, and Glu493, four Pi-Alkyl interactions with Leu523, His545, Phe605, and Ala636, two van der Waals with His492 and Gly550, and one Pi-sulfur interaction with Asp494. Lastly, conjugate **10** also made three hydrogen bonds with Ala440, Glu493, and Ala636, it was also involved in hydrophobic interactions with His492, Leu523, and His593. The molecular interactions and plausible binding modes of the competitive inhibitors in the binding pocket of urease are shown in Figure 6A. In the case of COX-2 enzyme, conjugate **8** made two hydrogen bonds with Arg106, one Pi-Cation interaction with Lys68, one Pi-sulfur interaction with Tyr101 as shown in Figure 6B.

**FIGURE 7:**
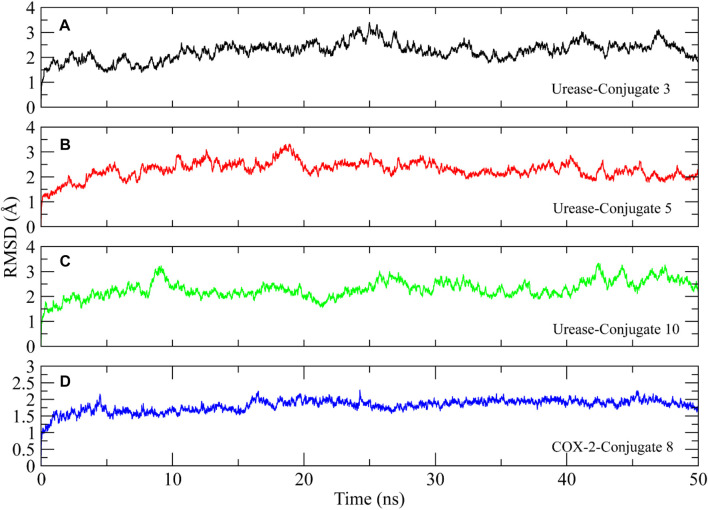
Root mean square deviation plots of the urease and COX-2 backbone atoms calculated during 50 ns simulation.

The stability of the protein-ligand complexes was assessed using various molecular dynamics simulation analyses. The backbone atoms RMSD of urease and COX-2 complexed with the conjugates were calculated to observe the structural stability of the complexes (Figure 7) (Sargsyan et al., 2017). It can be observed that all complexes of urease equilibrated at 5 ns and then the RMSD of conjugate **3** gradually increased to ∼3 Å at 25 ns and then decreased to ∼1.5 Å at 35 ns. It attained stability after 35 ns, in the range of ∼1.5–2.5 Å till the end of the simulation (Figure 7A). On the other hand, the RMSD of conjugate **5** showed deviations in the first 20 ns and then attained stability at ∼2–2.5 Å (Figure 7B). In case of conjugate **10**, the RMSD values remained in the range of ∼2–2.5 Å with some minor deviations towards the end of the simulation (Figure 7C). The RMSD of COX-2-Conjugate 8 complex indicated that the protein did not show deviations during the simulation and maintained the ∼1.5–2 Å range throughout the simulation (Figure 7D). The minor deviations in the trajectories indicated the stability of urease complexes.

**FIGURE 8:**
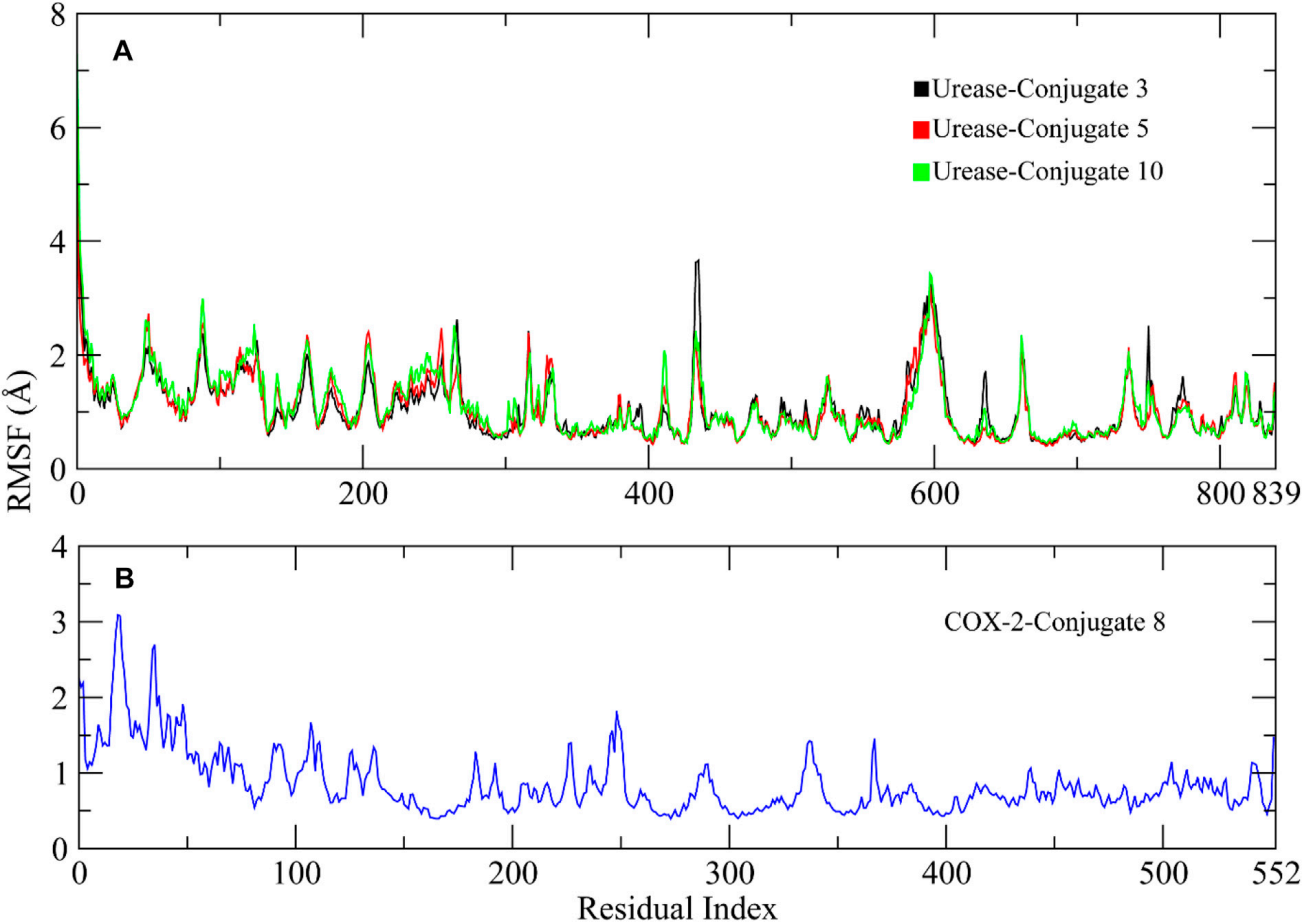
RMSF plots of the urease and COX-2 protein residues. **(A)** RMSF plots of Urease-Conjugate **3**, **5**, and **10** complexes. **(B)** RMSF plot of COX-2-Conjugate 8 complex.

RMSF analysis was performed to investigate the flexibility of residues in the protein (Martinez et al., 2015). Higher RMSF values indicate the loops and lower values indicate the rigidity of residues. The RMSF analysis showed similar plots for all complexes, with higher fluctuations observed in the starting residues due to the presence of loops at the N-terminal. The amino acid residues ranging from 50 to 60, 90 to 100, 110 to 125, 260 to 270, 420 to 530, 590 to 610, and 630 to 640 showed major fluctuations, indicating the presence of loops. Other portions of the protein remained rigid during the simulation except for the Conjugate **3** complex which showed some major fluctuation in residues 420 to 430 compared to other complexes (Figure 8A). The RMSF plot of COX-2 indicated that the residues remained rigid and did not show major fluctuations during simulation (Figure 8B).

**FIGURE 9:**
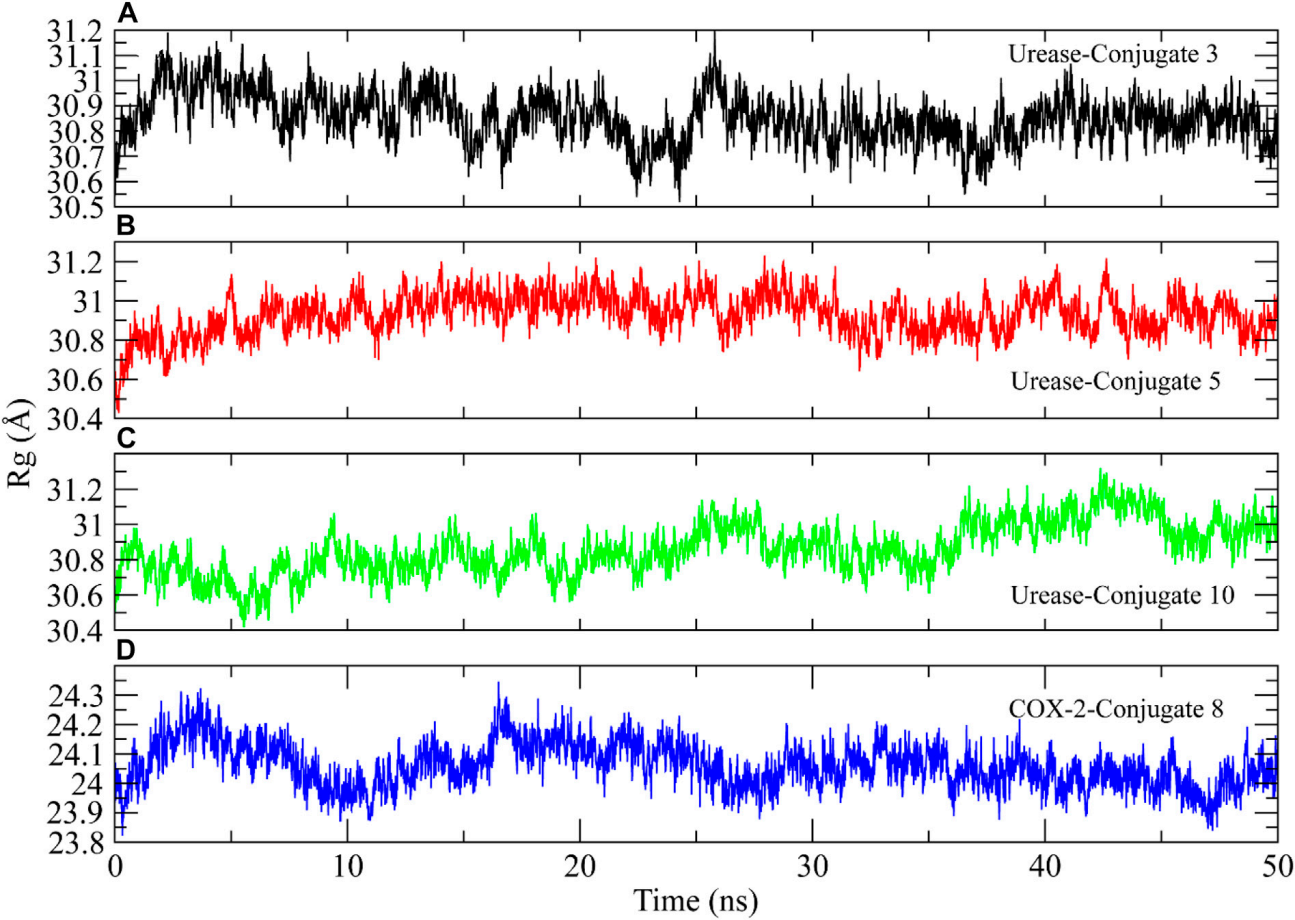
The radius of gyration calculation to analyze the compactness of urease and COX-2 protein structures during simulation.

Radius of Gyration (Rg) analysis was performed to assess the structural compactness of the urease proteins when bound to the conjugate (Lobanov et al., 2008). The lower Rg values indicate the structure stability while higher Rg values show distortions in the structure during simulation. The Rg plots of the complexes showed that the Rg values maintained a range of ∼30.08–31 Å after being equilibrated at 5 ns. The Rg value of conjugate 10 decreased to ∼30.06 Å at 35 ns and then again attained the previous range at 40 ns. The stable Rg values indicated that the protein structures remained compacted during simulation when bound to these conjugates (Figures 9A–C). Similarly, the Rg of COX-2 protein showed deviations in the range of ∼24–24.4 Å till 15 ns and then attained stability in the range of ∼24 Å till the end of the simulation (Figure 9D).

The physicochemical and ADMET (absorption, distribution, metabolism, excretion, and toxicity) properties of the sulfa drugs were analyzed. The compounds were within the allowed ranges for the ADMET characteristics. The cutoff values for the ADMET parameters were as follows: “QPlogHERG” (<-5), “QPlogPo/w" (−2.0–6.5), “QPlogBB” (−3.0 to 1.2), “QPPCaco” (<25 poor, >500 great), and “QPlogKhsa” (−1.5 to 1.5) (Tahir et al., 2018; Ashraf et al., 2022). The physicochemical and ADMET properties of the compounds are presented in Table 3. All the conjugates under investigation were within the octanol/water partition coefficient’s allowable range. The expected cell permeability and brain/blood partition coefficient were found to be satisfactory in this manner. However, the compounds demonstrated somewhat higher anticipated IC_50_ values for blocking HERG K+ channels.

**TABLE 3:** The physicochemical and ADMET properties of the conjugates.

Conjugates	MW	HBD	HBA	QPlogPo/w	QPlogHERG	QPPCaco	QPlogBB	QPlogKhsa
**3**	384.44	3	7	2.437	−6.444	234.441	−1.608	0.032
**4**	479.55	2	9	3.974	−7.326	386.293	−1.638	0.469
**5**	479.55	2	9	3.974	−7.326	386.293	−1.638	0.469
**6**	479.55	2	9	3.974	−7.326	386.293	−1.638	0.469
**7**	479.55	2	9	3.974	−7.326	386.293	−1.638	0.469
**8**	479.55	2	9	3.974	−7.326	386.293	−1.638	0.469
**9**	479.55	2	9	3.974	−7.326	386.293	−1.638	0.469
**10**	426.48	3	5	3.417	−6.688	58.155	−2.454	0.581

## 4 Conclusion

A bio-oriented drug synthesis is a promising approach for the development of new drugs and it can offer several advantages over traditional chemical synthesis, including increased selectivity, reduced waste, and lower costs. The present work reports the designed, successfully synthesized, and characterization of eight conjugates by coupling the naproxen with sulfa drugs which contain biologically important acetamide and sulfonamide scaffolds. The naproxen-sulfa drug conjugates were synthesized in good yield (68.7%–77.6%) from a one-step coupling reaction of naproxen and various substituted-sulfa drugs, and characterized by spectroscopic techniques. For urease, the best inhibitor among them was achieved for conjugate **10** (naproxen-sulfaguanidine), with a KI value of 3.56 µM. Conjugate **8** showed the highest inhibition effect among others against COX-2, with 75.4% inhibition which is comparable with celecoxib used as referenced COX-2 inhibitor. The binding stability of the competitive conjugates with urease and COX-2 revealed that the complexes did not show major deviations and remained compact during the MD simulation. Additionally, predicted ADMET properties shows that the synthesized naproxen-sulfa drug conjugates have drug-like properties which might show low toxicity, and adverse effects on performing *in vivo* assays. Furthermore, the *in silico* analysis showed that conjugates (3, 5, and 10) are binding at urease active site as determined in enzyme kinetics assays while Conjugate 8 is binding at COX-2 active sites.

## Data Availability

The original contributions presented in the study are included in the article/Supplementary Material, further inquiries can be directed to the corresponding authors.
