# Supplementary material for: Exploring the potential of propanamide-sulfonamide based drug conjugates as dual inhibitors of urease and cyclooxygenase-2: biological and their in silico studies
Source: Front Chem. 2023 Aug 3;11:1206380. doi: 10.3389/fchem.2023.1206380 (PMC10434765; doi:10.3389/fchem.2023.1206380)
Supplement: Supplementary file 1 [file DataSheet1.docx]

**Supplementary Data**

**Exploring the potential of propanamide-sulfonamide based drug conjugates as dual inhibitors of urease and cyclooxygenase-2: Biological and their in silico studies**

Saghir Ahmad ^1, 2^, Muhammad Abdul Qadir ^1^, Mahmood Ahmed ^3*^, Muhammad Imran ^4^, Numan Yousaf ^5^, Tanveer A. Wani ^6^, Seema Zargar ^7^, Ijaz Ali ^8^, Muhammad Muddassar ^5**^

^1^ School of Chemistry, University of the Punjab. Lahore 54590, Pakistan

^2^ Department of Microbiology, Immunology and Cancer Biology, School of Medicine, University of Virginia, Charlottesville, Virginia USA

^3^ Department of Chemistry, Division of Science and Technology, University of Education, College Road, Lahore-Pakistan

^4^ KAM School of Life Sciences, FC College (A Chartered University) Lahore- Pakistan

^5^ Department of Biosciences, COMSATS University Islamabad, Park Road, Islamabad- Pakistan

^6^ Department of Pharmaceutical Chemistry, College of Pharmacy, King Saud University, P.O. Box 2457,Riyadh 11451, Saudi Arabia

^7^ Department of Biochemistry, College of Science, King Saud University, P.O. Box 222452,Riyadh 11451, Saudi Arabia

^8^ CAMB, Gulf University for Science and Technology, Kuwait

**Running title:** Naproxen-sulfa drugs conjugates dual inhibitors of urease and cyclooxygenase-2

To whom correspondence should be addressed

Mahmood Ahmed, PhD

[mahmoodresearchscholar@gmail.com](mailto:mahmoodresearchscholar@gmail.com)

Muhammad Muddassar, PhD

[mmuddassar@comsats.edu.pk](mailto:mmuddassar@comsats.edu.pk)

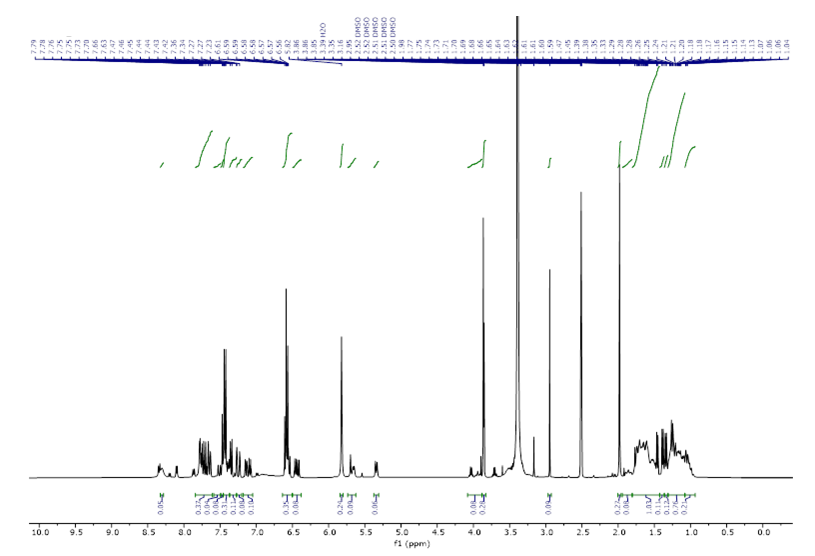


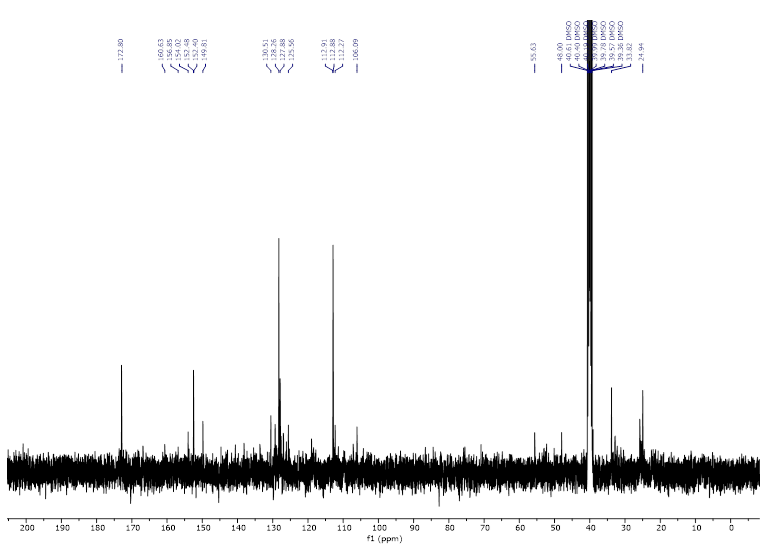


NMR spectra of (S)-2-(6-methoxynaphthalen-2-yl)-N-(4-sulfamoylphenyl)propanamide (3)

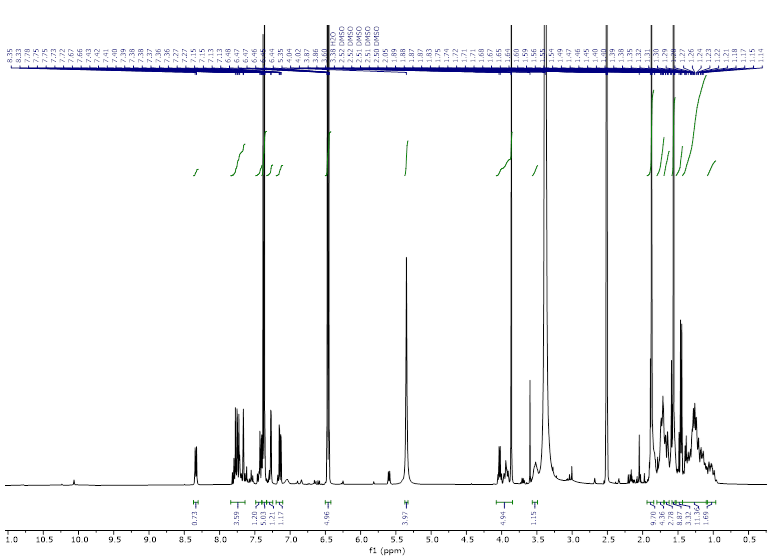


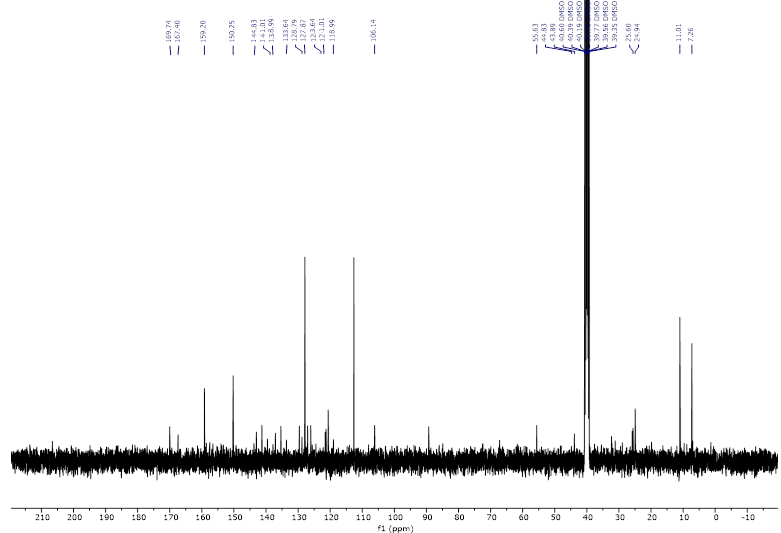


NMR spectra of (S)-N-(4-(N-(3,4-dimethylisoxazol-5-yl)sulfamoyl)phenyl)-2-(6-methoxynaphthalen-2-yl)propanamide (4)

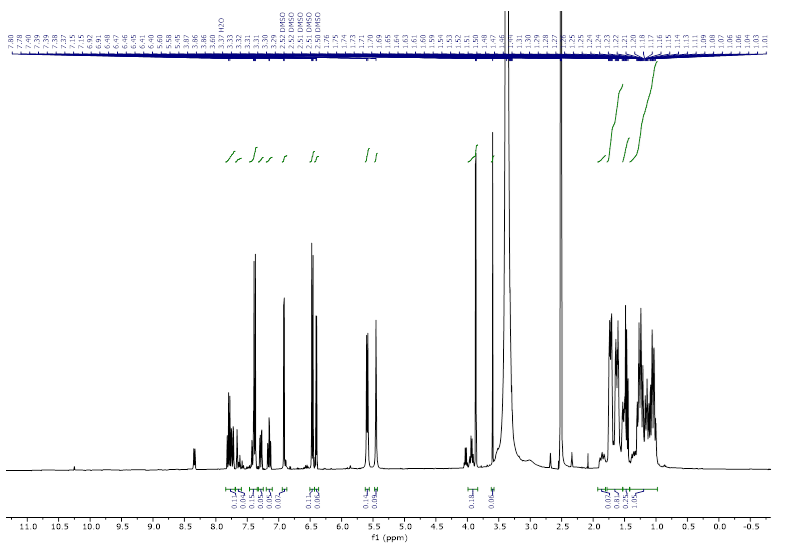


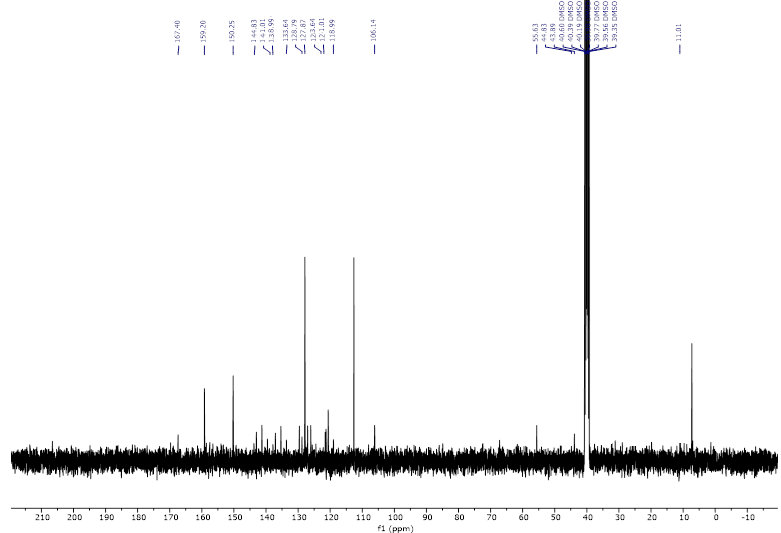


NMR spectra of (S)-2-(6-methoxynaphthalen-2-yl)-N-(4-(N-(thiazol-2-yl)sulfamoyl)phenyl)propanamide (5)

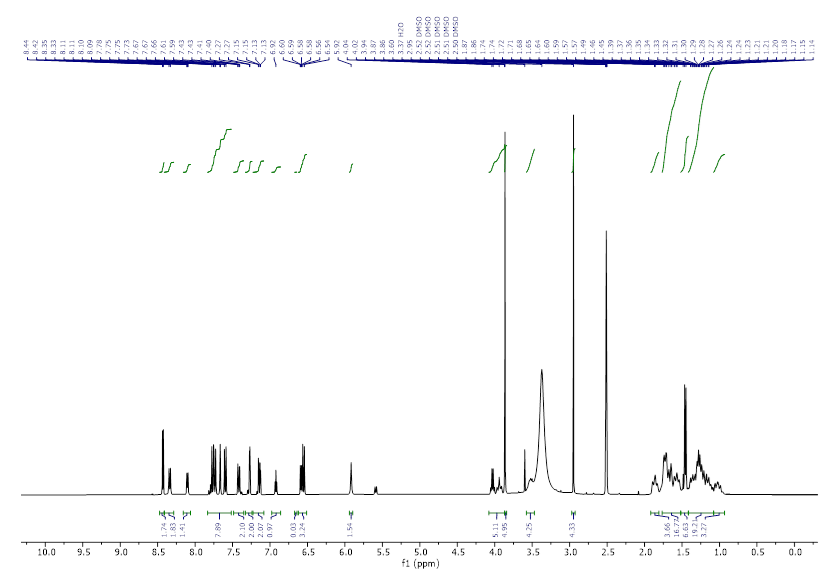


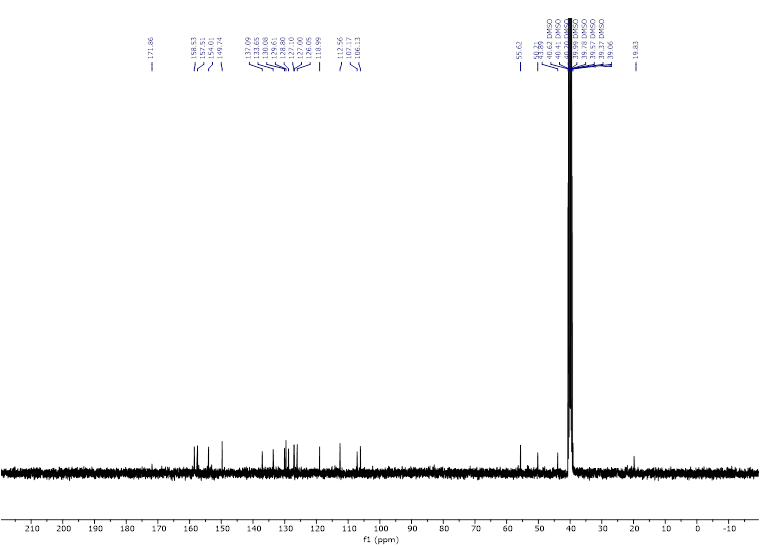


NMR spectra of 2.1.2.4. (S)-2-(6-methoxynaphthalen-2-yl)-N-(4-(N-(pyrimidin-2-yl)sulfamoyl)phenyl)propanamide (6)

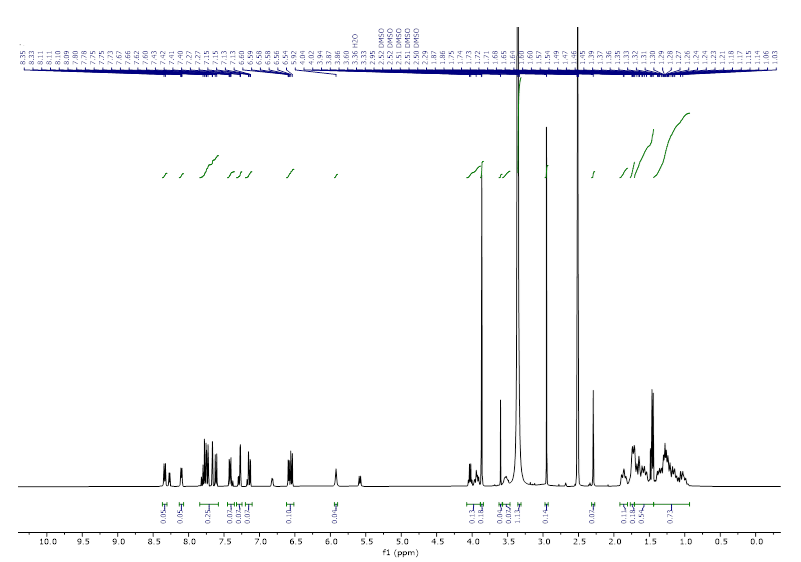


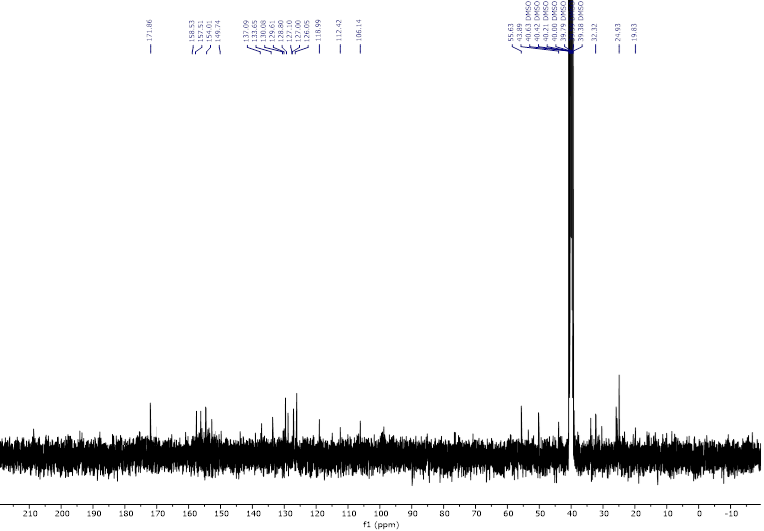


NMR spectra of 2.1.2.5. (S)-2-(6-methoxynaphthalen-2-yl)-N-(4-(N-(4-methylpyrimidin-2-yl)sulfamoyl)phenyl)propanamide (7)

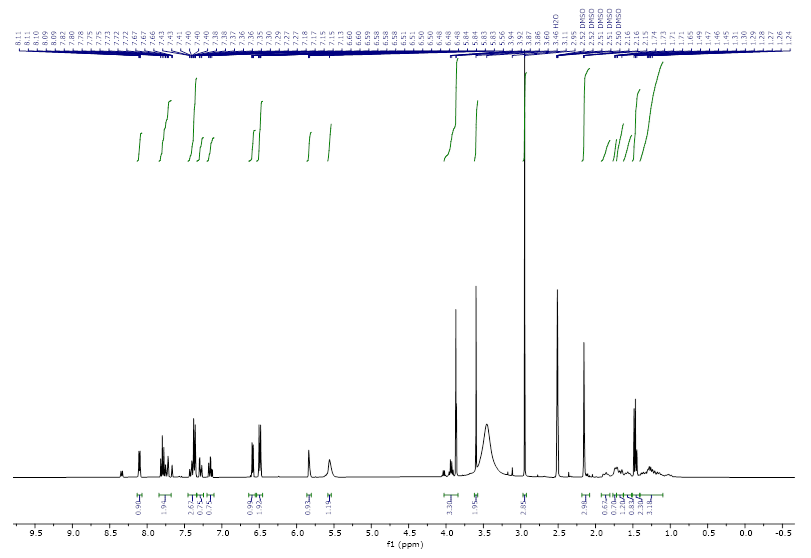


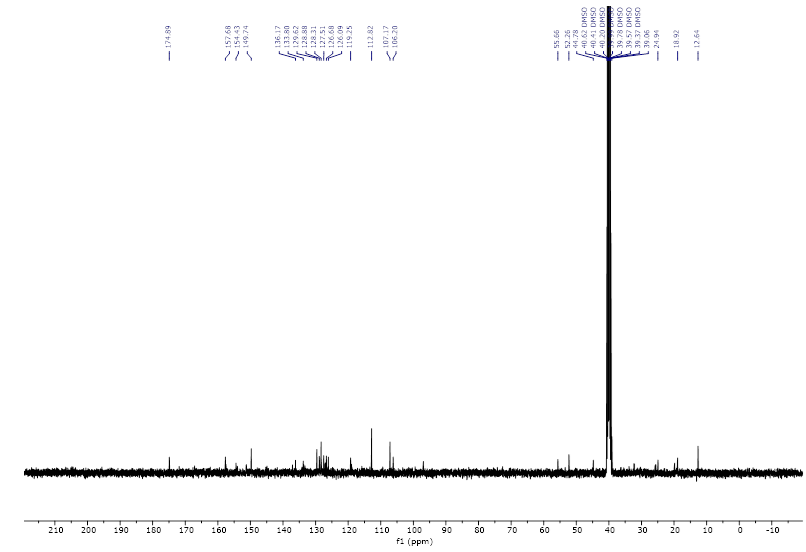


NMR spectra of2.1.2.6. (S)-2-(6-methoxynaphthalen-2-yl)-N-(4-(N-(5-methylisoxazol-3-yl)sulfamoyl)phenyl)propanamide (8)

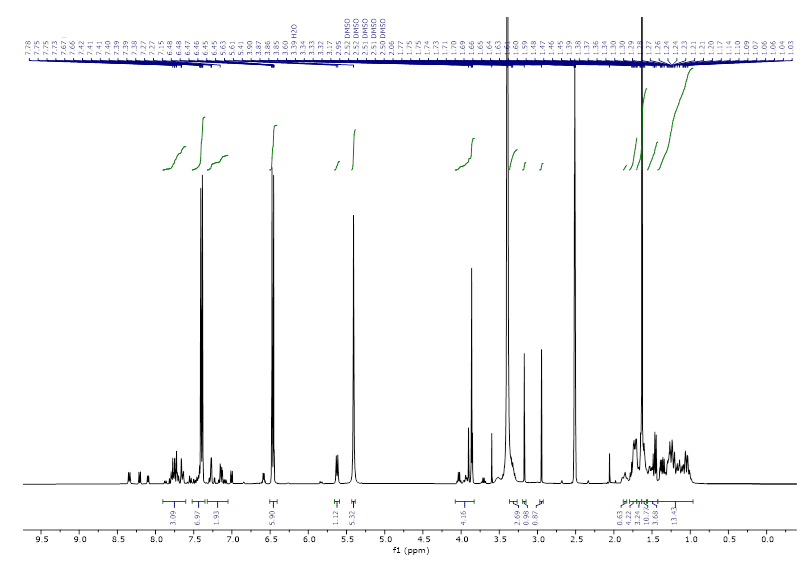


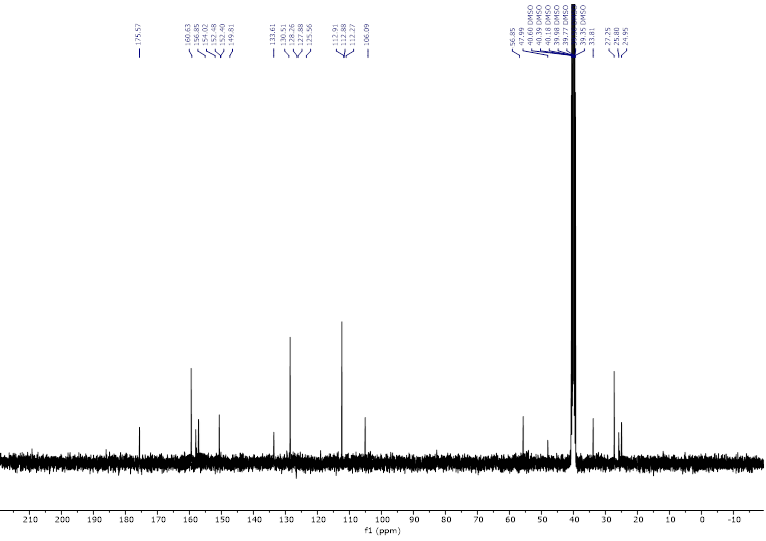


NMR spectra of (S)-N-(4-(N-acetylsulfamoyl)phenyl)-2-(6-methoxynaphthalen-2-yl)propanamide

(9)

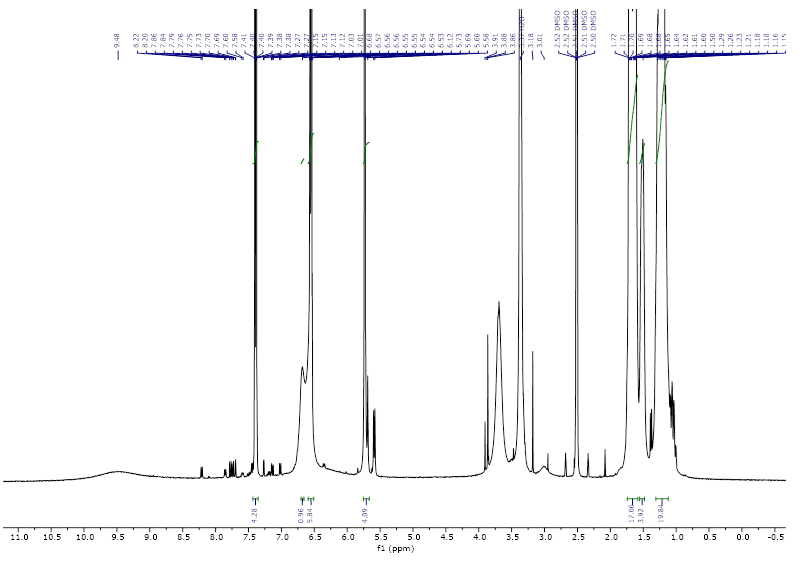


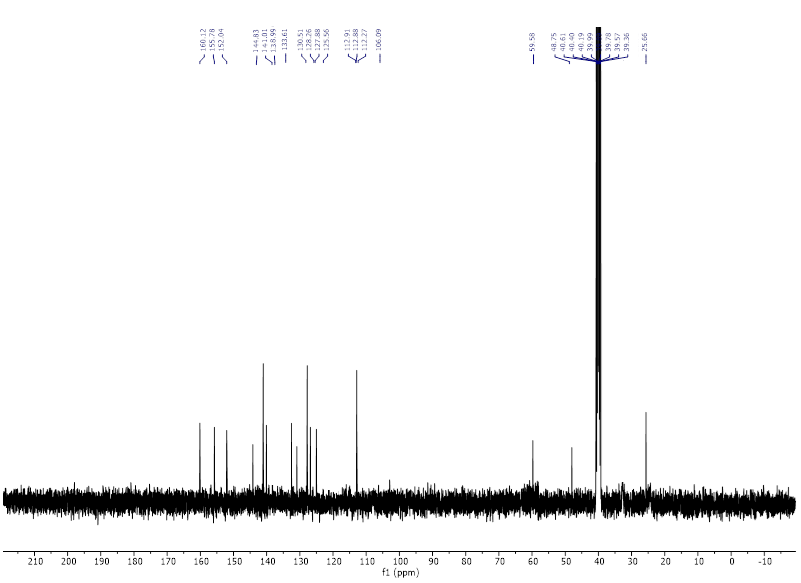


NMR spectra of 2.1.2.8. (S)-N-(4-(N-carbamimidoylsulfamoyl)phenyl)-2-(6-methoxynaphthalen-2-yl)propanamide (10)
